# Psychometric properties of the Albanian version of the Orofacial Esthetic Scale: OES-ALB

**DOI:** 10.1186/s12903-015-0083-x

**Published:** 2015-08-26

**Authors:** Venera Bimbashi, Asja Čelebić, Gloria Staka, Flurije Hoxha, Sanja Peršić, Nikola Petričević

**Affiliations:** 1 Department of Prosthodontics, Dental School, Faculty of Medicine, University of Prishtina, Prishtina, Kosovo; 2 Department of Prosthodontics, School of Dental Medicine, University of Zagreb and Clinical Hospital Centre, Zagreb, Croatia; 3 Department of Prosthodontics, Dental School, Faculty of Medicine, University of Prishtina and University Dentistry Clinical Center of Kosovo, Prishtina, Kosovo; 4 Department of Prosthodontics, School of Dental Medicine, University of Zagreb, Zagreb, Croatia

## Abstract

**Abstract:**

The aim was to adapt the Orofacial Esthetic Scale (OES) and to test psychometric properties of the Albanian language version in the cultural environment of the Republic of Kosovo.

**Methods:**

The OES questionnaire was translated from the original English version according to the accepted techniques. The reliability (internal consistency), and validity (construct, convergent and discriminative) were tested in 169 subjects, test-retest in 61 dental students (DS), and responsiveness in 51 prosthodontic patients with treatment needs (PPTN).

**Results:**

The corrected item correlation coefficients of OES-ALB ranged from 0.686 to 0.909. The inter-item correlation coefficient ranged between 0.572 and 0.919. The Cronbach’s alpha was 0.961 and IIC 0.758. Test- retest was confirmed by good ICCs and by no significant differences of the OES scores through the period of 14 days without any orofacial changes (*p* > 0.05). Construct validity was proved by the presence of one-factor composition that assumed 79.079 % of the variance. Convergent validity showed significant correlation between one general question about satisfaction with orofacial esthetics and the OES summary score, as well as between the sum of the 3 OHIP-ALB49 questions related to orofacial aesthetics and the OES summary score. Discriminative validity was confirmed with statistically significant differences between DS, prosthodontic patients without treatment need and PPTN (*p* < 0.01). Responsiveness was confirmed by a significant increase of OES scores after PPTN patients received new fixed partial or removable dentures (*P* < 0.001).

**Conclusion:**

The results proved excellent psychometric properties of the OES-ALB questionnaire in the Republic of Kosovo.

## Background

Pleasant facial appearance plays an essential role in community interactions. It may influence courage achievement and success in relationships, self-confidence in performance, better opportunities, better personality evaluations and prospective employment. However, what one culture understands as deficient another one may find it attractive. Esthetics is not absolute; it is extremely individual and subjective. Facial attractiveness correlates with self-esteem and it has been equally important for both, men and women [1, 2].

One of the main reasons why individuals ask for dental treatment is an improvement of their dental aesthetics. Some studies revealed that self-confidence and quality of life were higher in patients who were pleased with their dental treatment [3]. Dentofacial esthetics has been in addition associated with other common concepts of welfare [4, 5]. However, dentists have been far more serious in a perception of esthetics than dental patients or general population [6–17].

As lower third of the face has a major impact on perception of dentofacial esthetics, orthodontists, surgeons, restorative dentists and prosthodontists have an exceptional occasion to satisfy patients’ esthetic requirements by improving deficient dental and facial proportions and forms [18–20].

Well-being measurement scales have been fast upwards spots in medical and dental research [21–23]. However, such instruments have various uses, such as assessing perceived health or disease in population surveys, monitoring psychosocial aspects in individual patient care, measuring outcomes in clinical trials, and collecting data for cost-utility analyses [22–24]. One of the most widely accepted instruments for measurement of oral health-related quality of life (OHRQoL) is the questionnaire with excellent cross-culturally psychometric properties: the Oral Health Impact Profile (OHIP) [25]. Wong et al. recommended a new short form of the instrument, the OHIP-esthetic questionnaire, designed mostly for measuring changes of dental esthetics by teeth whitening [26]. Mehl et al. demonstrated that the OHIP-esthetics did not show satisfactory psychometric properties for appraising esthetical dental impairment, and they suggested the development of a more specific tool [27]. The new questionnaire: the orofacial esthetic scale (OES) has been recently developed in Sweden by Pernilla Larsson and co-authors [28]. The OES represents one-dimensional instrument consisting of eight items for measuring self-reported orofacial esthetics. Psychometric properties have been documented [29–33]. The original evaluation scale ranged from 0 to 10, but Croatian authors suggested using the 5 point Likert scale [31]. Revision of the instrument in new typical cultural context is not a straight forward concern. The original instrument must be translated and accommodated according to the accepted techniques, and the instrument needs to show adequate psychometric properties [34, 35].

At present, Kosovo has the youngest population in Europe with 53 % of the population being under the age of 29.5. Its long, troubled history and its wealthy culture are associated to loads of different empires prominent in the region over the centuries. The culture is an eclectic mix of ethnicities, including a number of separate languages, traditions and religions [36]. In the Republic of Kosovo, ethnic Albanians form the majority of a population, with Albanian being the country’s official language; though, no Albanian version of the OES is available so far.

The purpose of this study was to develop the Albanian language version of the OES questionnaire and to evaluate its psychometric properties among Albanian population in the Republic of Kosovo.

## Methods

### Participants

A sample of 169 subjects, aged from 19 to 86 years participated in this study. The study was approved by the Ethics Committee of the University Dentistry Clinical Center of Kosovo. The written consent was obtained from each subject after explanation of the aim of the study. The sample was divided into three groups. One convenience group comprised prosthodontics patients (PP, *n* = 57) with no treatment need. They had prosthetic restorations not older than one year and were satisfied. Twenty-seven of them had fixed partial dentures (FPD’s) and 30 had removable dentures (RD’s). The second convenience group included prosthodontics patients with treatment need (PPTN, *n* = 51), they came to a dental office asking for treatment. A specialist of prosthodontics assessed that 31 patients needed FPD’s and 20 patients needed RD’s. The two sample groups (PP and PPTN) were selected at the Department of Prosthodontics, School of Dental Medicine, University of Prishtina and Private Dental Clinic GS, Prishtina in Kosovo. The third consecutive group comprised of dental students (DS, *n* = 61) with natural teeth, without any need for dental treatment, and without orthodontic or dentofacial anomalies (malocclusion, anomalies of jaw size, jaw relationship, dental arch relationship, tooth position, etc.).

### Orofacial esthetic scale translation

The OES English language version was translated into the Albanian language according to the conventional methodology, following the procedure already used in previous adaptation and validation studies in other countries [31–33].

The English version with 8 items was translated by a qualified translator, with excellent knowledge of dental vocabulary. For the translation of some expressions in the Albanian language, three other dentists with excellent proficiency in the English language were also included. The translated version was edited by two other dentists, with excellent comprehension of English language (Dental School, Faculty of Medicine, University of Prishtina). The translation was done individually and in the end the final editing was integrated into the definitive one. Further, the final version was back-translated into the English language by an independent qualified translator, together with three other dentists with excellent comprehension of English language. The final version, translated back to the English language, was evaluated independently by one native English language speaker and two professors from the Dental School, University of Zagreb, with excellent expertise in English. They confirmed that there was no significant differences from the back translated and the original version. The final translation was considered to be satisfactory for its further use.

### OES-ALB cross cultural adaptation

Prior to this study, the clarity of the OES in the Albanian language was tested in 30 prosthodontic patients (age range 27–63 years) who were not involved in the study. Feedback regarding any problems of understanding and answering the questionnaire was obtained and addressed. Consequently for avoiding misunderstanding of overall face aesthetic, as face wrinkles, shape of nose or eyes, as the OES has been designed exclusively for assessing the esthetics of the lower third of the face and teeth, the items “Your facial appearance” and “Appearance of your facial profile” were slightly revised and specified as “Appearance of the lower third of your face” and “Appearance of the lower third of your facial profile”. The original OES version utilized the 11 point rating scale (10 = very satisfied and 0 = very dissatisfied) [28]. As suggested in the Croatian study [31], the Likert 5 point scale (1 = unsatisfactory; 5 = excellent), and also applied in the Chinese OES version [32] was used in this study because of the traditional evaluation in primary and secondary schools in Kosovo uses the 5 point scale (1 = unsatisfactory; 5 = excellent). The first seven items face frontally, profile, mouth (lips, visible of teeth, smile line), tooth position, tooth form, tooth color and gum indicate to exclusive esthetic components and scores of these first seven items can comply to form an OES summary score. The last eighth item appraises respondents general satisfaction with their esthetic and together with other first seven components can be combined to form an OES total summary score. The lower scores characterized dissatisfaction with orofacial esthetics and in contrary, higher scores represented higher satisfaction. Besides the OES questionnaire items, the subjects also answered three questions from the Albanian version of the OHIP-49 questionnaire which were related to esthetics (questions number 3, 22 and 31) [35]. The PP and PPTN groups were interviewed and the DS group self-administered the OES questionnaire but, they were supervised by three dentists.

### Statistical analysis

The reliability (internal consistency and test-retest), validity (construct, convergent, discriminative) and responsiveness of the OES-ALB were assessed.

### Reliability

Two categories of reliability were measured; the internal consistency and test–retest reliability (the consistency of the scores through a reasonable period of time). The internal consistency was tested by using the average inter-item correlation and Cronbach’s alpha coefficient [37]. Average inter-item correlation should be more than 0.40. According to guidelines, Cronbach’s alpha values > 0.75 correspond to excellent outcome, values from 0.40 to 0.75 are considered satisfactory and the value of < 0.40 are considered as poor outcome [37]. Test–retest reliability was assessed as temporal stability, which was tested by calculating the interclass correlation coefficient (ICC) using the one-way analysis of variance [38]. The test-retest reliability was performed only in the group of Dental Students, who filled-in the OES questionnaire twice within a period of 14 days between trials. Participants did not receive any dental or oral treatment during the observed period. The values of ICC <0.40 indicated poor interclass correlation, 0.41- 0.60 moderate, 0.61 – 0.80 good and the values > 0.80 indicated excellent interclass correlation [38].

### Validity

The construct, convergent, and discriminative validities were tested [39]. All participants were included. The exploratory factor analysis (EFA) was used for assessing the number of factors of the OES-Alb. The Kaiser-Mayer-Olkin (KMO) test, Bartlett’s test of sphericity and the scree plot were used [40, 41]. The eigenvalue above 1 was set as criteria for factor with a drawl; also significant factor loadings > 0.30 were defined. The convergent validity was tested by Spearman’s rank correlation between a self-reported general satisfaction with orofacial esthetics and the OES summary score. It was also tested by the Spearman’s rank correlation between the OES summary score and the sum of three questions from the OHIP-ALB49 related to esthetics (items 3, 22 and 31) [35]. Discriminative validity assumes that unrelated measures are in reality not related. It was predicted that dental students without orofacial anomalies would have better esthetics than prosthodontics patients with a treatment need. The discriminative validity was tested by comparing the OES total summary score between the three sample groups (PP, PPTN, and DS) using the one-way analysis of variance and the Sheffe post-hoc tests.

### Responsiveness

It was tested in 51 prosthodontic patients with treatment requirements [42]. They completed the questionnaire twice; prior to treatment and a month later after they had received new dentures. Thirty-one of them received fixed partial dentures (FPDs) and twenty of them received removable denture (RDs). A month period was considered sufficient for adaptation to new dentures and new esthetical appearance. The difference of the OES total summary scores between the baseline and the follow-up was tested using the paired t-test and by calculating the effect size and the standardized response mean [43]. According to Cohen the effect size of > 0.80 is considered large, 0.50 moderate and 0.20 small [44]. The standardized effect size was determined using the formula [44]:

$$ Mean\left(baselineOESscore-followupOESscore\right)/StandarddeviationofthebaselineOESscore $$

Statistical analyzes were computed using MS Excel (Microsoft Office, Windows 2007, USA) and SPSS 19 for Windows (SPSS Inc., Chicago, Illinois, USA) software.

## Results

### Sample overview

Overview of groups of participants, their age, gender, data collection methods, sampling strategies and research purposes are presented in details in Table 1.

**Table 1:** Sample overview (number, age, and gender), sampling strategies, research purpose and data-collection methods – OES in Albanian language

Sample	Sample type	N	Age mean (SD)	Age range	% women	Type of investigation
Prosthodontic Patients - PP (*n* = 57)	Convenience	57	49.92 (14.52)	20-86	49.12	Convergent Validity
Fixed Partial Dentures (FPD’s) (*n* = 27)						Internal Consistency
						Discriminate Validity
Removable Dentures Wearers (RDW’s) (*n* = 30)						
Prosthodontic Patients with a Treatment Need- PPTN (*n* = 51)	Convenience	51	49.50 (16.13)	19-73	50.98	Convergent Validity
Fixed Partial Dentures (FPD’s) (*n* = 31)						Discriminate Validity
						Internal Consistency
						Responsiveness
Removable Dentures Wearers (RDW’s) (*n* = 20)						
Dental Student – DS	Consecutive	61	22.13 (0.46)	21-23	60.65	Convergent Validity
						Internal Consistency
						Test-Retest Reliability
Natural Teeth (*n* = 61)						Discriminate Validity

^PP, PPTN^ Prosthodontic patients - Department of Prosthodontics, School of Dental Medicine, University of Prishtina and Private Dental Clinic GS, Prishtina, Kosovo; interviewed

^DS^ Dental Students -Natural teeth, School of Dental Medicine, University of Prishtina; self-administered questionnaire - supervised

### Reliability

The corrected item-total correlation ranged from 0.686 to 0.909. The lowest coefficient was found for the seventh question “Your gum’s appearance?” and the highest coefficient for the first item: “Your facial appearance?”. If items were deleted one by one, the Cronbach’s alpha would not increase and it ranged between 0.952 and 0.956 (Table 2). The correlations between items are presented in Table 3. The weakest correlation was found between items “Appearance of your rows of teeth?” and “Your gum’s appearance?”. The highest inter-item correlation was found between “Appearance of your facial profile?” and “Your facial appearance?”(Table 3). Internal consistency of the OES-ALB showed excellent results based on average inter-item correlation of 0.758 and Cronbach’s alpha value of 0.961. The test-retest reliability was performed in the DS group (Table 4). The ICCs were appropriate, and there were no significant differences, either for each of the items, or for the OES total summary score (NS = not significant, *p* > 0.05) between the two occasions (two week period).

**Table 2:** Results obtained for the OES – ALB set in validation sample

OES item	Mean	SD	Corrected Item-Total Correlation	Cronbach’s Alpha if Item Deleted	Factor Loading
1. Your facial appearance	3.49	1.12	0.909	0.952	0.941
2. Appearance of your facial profile	3.44	1.10	0.848	0.956	0.890
3. Your mouth’s appearance (smile, lips and visible of teeth)	3.44	1.17	0.887	0.953	0.918
4. Appearance of your rows of teeth	3.49	1.29	0.849	0.956	0.882
5. Shape/form of your teeth	3.48	1.36	0.892	0.953	0.916
6. Color of your teeth	3.44	1.32	0.874	0.954	0.901
7. Your gum’s appearance	3.76	0.97	0.686	0.964	0.748
8.Overall, how do you feel about of your face, your mouth and your teeth	3.50	1.06	0.875	0.954	0.908

**Table 3:** Inter-Item Correlation matrix of OES-ALB

Item	E1	E2	E3	E4	E5	E6	E7	E8
E1	1.000							
E2	.919	1.000						
E3	.883	.810	1.000					
E4	.753	.688	.770	1.000				
E5	.792	.744	.793	.871	1.000			
E6	.772	.724	.766	.831	.908	1.000		
E7	.674	.619	.687	.572	.583	.618	1.000	
E8	.852	.802	.808	.770	.796	.774	.638	1.000

**Table 4:** Test-retest reliability for each item and summary score OES-ALB, Dental Student (DS) group

Item	Mean difference	Test -Retest (ICC)	95 % CI of the Difference	T	P
E1	0.03	0.887	−0.05 ± 0.11	0.814	0.419 NS
E2	−0.05	0.820	- 0.17 ± 0.07	−0.830	0.410 NS
E3	0.03	0.856	−0.07 ± 0.14	0.629	0.532 NS
E4	−0.08	0.870	−0.19 ± 0.03	−1.524	0.133 NS
E5	0.05	0.891	−0.05 ± 0.15	1.000	0.321 NS
E6	−0.07	0.887	−0.16 ± 0.03	−1.426	0.159 NS
E7	−0.08	0.826	−0.19 ± 0.03	−1.524	0.133 NS
E8	0.03	0.743	−0.09 ± 0.16	0.531	0.597 NS
OES Total Summary Score Test- Retest	−0.13	0.940	−0.48 ± 0.22	−0.747	0.458 NS

### Validity

Factor loadings for each item ranged between 0.748 and 0.941 (Table 2). The Bartlett’s test of sphericity was 1646.154 (df =28, *P* < 0.001) and Kaiser – Meyer - Olkin (KMO) test was 0.921, more than critical value 0.60. Exploratory factor analyzes exposed the one-factor structure on the basis of the eingenvalue >1 and assumed 79.079 % of the variance, confirming the one-dimensional model of the OES-ALB, as well as the scree plot. Convergent validity was confirmed by significant relationship between a self-reported general satisfaction with orofacial esthetics and the OES summary score using the Spearman’s rank correlation, as well as by statistically significant correlation between the sum of 3 questions from the OHIP-ALB related to esthetic and the OES summary score (Table 5). Discriminative validity was tested by comparison of the OES total summary scores between the three sample groups: PP, PPTN, and DS. One way ANOVA revealed statistically significant differences between the groups (*p* < 0.05) (Table 6).

**Table 5:** Convergent validity of the OES-ALB

Correlations					
			General satisfaction with esthetic	OES-ALB summary score	OHIP3 esthetic summary score
Spearman’s rho	General Satisfaction with Esthetic	Correlation Coefficient	1.000	0.888**	−0.654**
		Sig. (2-tailed)	.	0.001	0.001
		N	169	169	169
	OES-ALB Summary Score	Correlation Coefficient	0.888**	1.000	−0.714**
		Sig. (2-tailed)	0.001	.	0.001
		N	169	169	169
	OHIP3 Esthetic Summary Score	Correlation Coefficient	−0.654**	−0.714**	1.000
		Sig. (2-tailed)	0.001	0.001	.
		N	169	169	169

Spearman’s rank correlation; ** *p* < 0.01

**Table 6:** Discriminative validity of the OES-ALB; significance of the differences between the OES total summary scores between DS, PPTN and PP groups

Group	*N*	X	SD						
				F	P		PP	PPTN	DS
Prosthodontic Patients (PP)	57	30,86	4,14	194.09	<0.001	PP		*	*
Prosthodontic Patients with Treatment Needs (PPTN)	51	17,63	5,65			PPTN	*		*
Dental Students with Healthy Natural Teeth (DS)	61	34,11	4,01			DS	*	*	

(One-Way ANOVA, Sheffe post hoc) * *p* < 0.05

### Responsiveness

It was tested only in the PPTN group. The questionnaire was administrated twice; the first time prior treatment and the second time a month later after patients had received their prosthodontic restorations. As it was predicted, the significant OES score increase was found after the treatment. The mean change between the baseline and the after treatment total summary scores in the FPD group was 17.16 (SD =2.12) (*P* < 0.001) (Table 7). The effect size for the OES-ALB total summary score in the FPD group was large, 3.54. The scores also increased significantly in the RD group. The difference in the RD group between the baseline and the after treatment total summary score was 18.40 (SD =2.4) (*P* < 0.001) (Table 7). The effect size was 3.10.

**Table 7:** Responsiveness in prosthodontic patients with treatment need (PPTN), who received fixed partial dentures and removable partial dentures

	PPTN- fixed partial dentures			PPTN -removable dentures		
OES-ALB Items	Before treatment	After treatment	P	Before treatment	After treatment	P
	x ± SD	x ± SD		x ± SD	x ± SD	
E1	2.55 ± 0.85	4.42 ± 0.50	0.001	1.85 ± 0.81	4.15 ± 0.59	0.001
E2	2.55 ± 0.81	4.52 ± 0.51	0.001	1.90 ± 0.79	4.10 ± 0.55	0.001
E3	2.35 ± 0.84	4.71 ± 0.46	0.001	1.80 ± 0.89	4.05 ± 0.51	0.001
E4	2.16 ± 0.82	4.68 ± 0.48	0.001	1.60 ± 0.99	4.35 ± 0.49	0.001
E5	1.97 ± 0.84	4.71 ± 0.46	0.001	1.50 ± 1.00	4.30 ± 0.47	0.001
E6	2.03 ± 0.87	4.81 ± 0.40	0.001	1.55 ± 1.05	4.35 ± 0.49	0.001
E7	3.16 ± 0.58	4.16 ± 0.69	0.001	2.75 ± 0.85	3.90 ± 0.79	0.001
E8	2.52 ± 0.72	4.45 ± 0.51	0.001	2.10 ± 0.97	4.25 ± 0.55	0.001
OES Total Summary Score	19.29 ± 4.85	36.45 ± 2.73	0.001	15.05 ± 5.93	33.45 ± 3.53	0.001

*statistically significant difference; *p* < 0.001; FPD’s (df = 30)**;** RPD’s (df = 19)

## Discussion

This study was designed to adjust the OES questionnaire to the Albanian language version in the cultural domain of the Republic of Kosovo and to evaluate the psychometric properties of the OES-ALB. Prosthodontic patients should increase their orofacial esthetics after they receive new dentures. To obtain the appropriate instrument in the Republic of Kosovo we decided to translate the OES questionnaire into Albanian language according accepted principles and to test the translated psychometric properties of the OES questionnaire in the new cultural environment [34]. The evaluation, design and testing of psychometric properties of the OES in the Albanian language was made similar to the adaptation of the Croatian and the Chinese versions [31, 32]. Our administration technique was similar with strategies applied in Croatian, Chinese and German versions of the OES questionnaire [31–33]. The items were summed to obtain the OES-ALB total summary scores. Due to the traditional ratings in the primary and secondary schools in Kosovo: grades range from 1 to 5 (unsatisfactory to excellent); we used recommended 5-point Likert scale [31]. We also asked questions associated with oral aesthetics obtained in the OHIP-ALB49 (item 3, 22 and 31) [35]. The OHIP scores were rated from 0 = no problems to 4 = more often problems.

The previous studies showed that the OES questionnaire was one-dimensional instrument [28, 31–33] which has also been proved by the present study. The Cronbach’s alpha coefficients also showed excellent consistency for the OES-ALB [37].

The test-retest reliability was tested only in the student group, same as it was done in some previous studies [35]. We excluded the PP patients from test-retest because we assumed many drop-offs. We had already asked them to come for a recall visit one year after their treatment and we assumed that they will not come back in 2 weeks only to fill-in the questionnaire for the second time. The OES-ALB showed good test-retest results in the DS group, with no significant difference between the two occasions, both for the total summary score and for each of the items (*P* > 0.05), which was similar to the original and other OES adaptation studies [31–33].

The construct validity was measured by exploratory factor analysis; Kaiser–Meyer-Olkin (KMO) test and the factor loadings, which were much higher than the limits for the OES-ALB. The factor analyzes revealed one-dimensional questionnaire and explained 79.079 % of the variance confirming good construct [45].

Convergent validity was confirmed by significant relationship between self-reported general satisfaction with esthetics and the OES-ALB summary score, as well as by significant correlation between the sum of the 3 OHIP-ALB questions related to esthetics and the OES summary score. The results are also comparable with other studies concerned with the OHIP questionnaire adaptation studies [28–33, 46, 47].

Discriminative validity was tested by assessment of the significance of the differences of the OES-ALB total summary scores between three sample groups. The PPTN group was predicted to have the lowest esthetic outcome, which has been confirmed by the lowest scores in the present study (*p* < 0.05).

However, the responsiveness was tested only in the PPTN group, which comprised patients who needed both, FPDs and RDs. They filled-in the OES questionnaire prior to the treatment and a month later after they had received prosthodontic restorations and got well adapted to them. As predicted, we found statistically significant increase of the OES-ALB total summary scores for both, the FPD and the RD patients with the high effect size, as well as improvement of patients’ esthetics from prosthodontic therapy. The effect size was slightly higher in the FPD group than in the RD group.

Results obtained in the present study established excellent psychometric properties of the OES-ALB questionnaire and confirmed the possibility of its implementation in the cultural environment of the Republic of Kosovo.

It should be noted that this study included few limitations, which include relatively a small number of PP patients (as the study was done in a specific area of huge intellectual variety and range of society standards; however we tried to include both, urban and rural participants, different social groups and patients of different degree of education). The test-retest was done only in the DS group, so low educated and low-income participants of the same age were not included. Further, the OES scores were correlated only with self-rated esthetics and with 3 OHIP questions related to esthetics while the expert group ratings were not performed.

The strength includes pilot testing and all necessary steps done as recommended for a proper validation. Good psychometric properties of the OES-ALB will consequently allow further investigation concerning esthetic normative values in a general population of the Republic of Kosovo, as well as in specific populations and/or patient groups. It would be interesting to compare esthetic normative values between population living in cities and rural areas, young and old, different social groups, etc., mostly due to specific cultural and economic characteristics of the region. The adaptation OES-ALB will also enable a comparison of the results from Kosovo with the results obtained in more developed countries and specific cultures, as differences in norms and values across cultures may exist. The strengths of this study lies also in the fact that we established the sensitivity of the instrument to changes i.e., responsiveness, the instrument’s facility for detecting differences.

## Conclusion

The Albanian language version of the OES questionnaire, translated and adapted into a new cultural environment in the Republic of Kosovo showed excellent psychometric properties and was confirmed as the one-dimensional instrument. The study was conducted in a specific area of huge intellectual variety and a range of social standards. The psychometric properties of the OES-ALB are similar with other validated OES language versions.

## Abbreviations

OES: Oral esthetic scale
OES-ALB: Oral esthetic scale in Albanian
OHRQoL: Oral health related to quality of life
OHIP: Oral health impact profile
PP: Prosthodontic patients
PPTN: Prosthodontic patients with treatment need
NT: Natural teeth
FPD: Fixed partial denture
RD: Removable denture
ICC: Inter class correlation
